# Doxycycline-Eluting Core-Shell Type Nanofiber-Covered Trachea Stent for Inhibition of Cellular Metalloproteinase and Its Related Fibrotic Stenosis

**DOI:** 10.3390/pharmaceutics11080421

**Published:** 2019-08-19

**Authors:** Rengarajan Baskaran, Un-Jeong Ko, Enkhzaya Davaa, Ji Eun Park, Yixin Jiang, Junghan Lee, Su-Geun Yang

**Affiliations:** 1 Department of Biomedical Science, Inha University College of Medicine, Incheon 22332, Korea; 2 Inha Institute of Aerospace Medicine, Inha University College of Medicine, Incheon 22332, Korea

**Keywords:** electrospinning, coaxial nanofiber, fibrosis, trachea stenosis, doxycycline, MMP

## Abstract

In this study, we fabricated a doxycycline (doxy)-eluting nanofiber-covered endotracheal stent for the prevention of stent intubation-related tissue fibrosis and re-stenosis. The nanofiber was deposited directly on the outer surface of the stent using a coaxial electrospinning method to form a doxy-eluting cover sleeve. Poly(d,l-lactide) was used as the shell-forming polymer and dedicated drug release-control membrane. Polyurethane was selected as the drug-loading core polymer. The compositional ratio of the core to shell was adjusted to 1:0, 1:2, and 1:4 by changing the electro-spray rate of each polymeric solution and microscopic observation of nanofibers using scanning electron microscopy (SEM), transmission electron microscopy (TEM), and the fluorescence microscopy proved core-shell structure of nanofibers. The in vitro release study suggested that the release of doxy could be controlled by increasing the compositional ratio of the shell. The growth of HT1080 fibrosarcoma cells was inhibited by the 10% doxy-containing nanofiber. The real-time polymerase chain reaction (PCR) in HT1080 cells and xenografted tissue models indicated that the doxy-releasing nanofiber inhibited mRNA expression of metalloproteinases (MT1-MMP, MMP-2, and MMP-9). Overall, our study demonstrates that a doxy-eluting core-shell nanofiber stent can be successfully fabricated using coaxial electrospinning and displays the potential to prevent fibrotic re-stenosis, which is the most problematic clinical complication of tracheal stent intubation.

## 1. Introduction

Tracheal intubation is the placement of a conduit into the trachea to prevent airway obstruction [1,2,3]. Tracheal intubation is performed on patients during emergency situations such as respiratory failure, stroke, and coma in the clinic [4,5,6]. However, paradoxically, the trachea stent itself induces re-stenosis accompanied by granulomatosis from stent-induced inflammation and tissue damage [7]. Stent-induced inflammation causes infection, the development of tissue fibrosis, the formation of granulomatous tissues, and eventually the narrowing of the trachea duct, leading to stenosis [8,9,10]. Tracheal restenosis or fibrosis is the most common and serious complication of prolonged intubation [11]. Resection of over-grown tissues can be performed as a palliative treatment for patients. However, there is a lack of medical or surgical options to prevent stent-induced tissue fibrosis. 

Tracheal fibrosis is an inflammation-related hyperplastic tissue formation associated with structural changes in the extracellular matrix (ECM). Matrix metalloproteinase (MMP) levels are significantly higher in inflammatory tissue and cause fibrosis or stenosis in human organs [12,13]. Among them, MMP-2 and MMP-9 are predominantly expressed in inflammatory tissues [14]. Several inhibitors, including hydroxamates, carbamoyl phosphonates, and antibiotics, have been explored to down-regulate MMPs. However, the control of MMP expression has been unsuccessful in clinical trials, because of poor specificity to MMPs [15,16]. Interestingly, doxycycline (doxy), a broad-spectrum antibiotic, exhibits MMP inhibitory activity [17]. Doxy potentially inhibits the expression of MMP-2 and MMP-9 in inflammatory cells [18]. To date, only doxy has been approved by the United States Food and Drug Administration (FDA) as an MMP inhibitor [19]. In addition, doxy likely prevents infection-related inflammation and thus improves the clinical response to tracheal stent intubation. 

Electrospinning is a simple, flexible, cost-effective, and preferred technique for fabrication of nanofibers. Various polymers such as polyurethane (PU), poly(ε-caprolactone), poly(lactic-*co*-glycolic acid), and chitosan can be successfully electrospun into nanofibers. Electrospun nanofibers have extremely high surface-to-mass (or volume) ratios and porous structures with excellent interconnectivity [20,21]. The wide spectrum of polymer selection for electrospinning allows various application designs of nanofibers with desirable properties. Among the applications, drug-eluting stents coated with a polymeric nanofiber might be one of the most practical medical engineering applications. So far, various nanofiber systems have been studied for cardiac and gastrointestinal stents. Drug-eluting nanofiber stents have been tested for local tumor chemotherapy. A paclitaxel-eluting nanofiber-covered stent demonstrated tumor growth inhibition in preclinical and clinical trials [22,23]. In this study, we developed a doxy-eluting nanofiber-covered stent by using electrospinning technology for the treatment of tracheal stenosis.

Based on previous reports, we postulated that a doxy-eluting nanofiber stent could be effective for treating tracheal stenosis and fabricated a doxy-loaded core-shell nanofiber with different core-shell compositional ratios (1:0, 1:2, and 1:4). The morphology of the nanofiber was characterized using scanning electron microscopy (SEM), transmission electron microscopy (TEM), and fluorescence microscopy. The amount of released doxy from each nanofiber stent was estimated. We also performed an in vitro cell study and estimated the MMP expression level in HT1080 fibrosarcoma cells after treatment with nanofiber stents. 

## 2. Materials and Methods

### 2.1. Materials

Doxy, tetrahydrofuran, and N, N-dimethylformamide were purchased from Sigma-Aldrich (St. Louis, MO, USA). Stent grafts for animal study and electrospinning were kindly supplied from S&G Biotech Inc. (Gyeonggi-do, Korea). The polyurethane (Pellethane 2363-80A, Lubrizol^®^, The Lubrizol Co., Wickliffe, OH, USA) was kindly supplied by Taewoong Medical Co. (Gyeonggi-do, Korea). Poly(d,l-lactide) (PDLLA, RESOMER^®^ R 202 H) was purchased from Evonik Health Care (Essen, Germany). Methanol and acetonitrile were used as HPLC-grade and were purchased from J.T. Baker (Fisher Scientific Co., Hampton, NH). Distilled water was from Milli Q (Millipore Co., Miliford, MA, USA). All other chemicals of analytical reagent grade were used without further purification.

### 2.2. Preparation of Nanofiber Solution 

A single-layer nanofiber was fabricated using a polyurethane (PU) polymer with doxy. Doxy was dissolved in a mixture of *N*, *N*-dimethylformamide (DMF) and tetrahydrofuran (THF) solution (2:1). Then, 30 mg of PU polymer (10% *w*/*w*) was added to the above solution and vortexed until the solution was clear. This solution was used as the core in the co-axial system. For the shell layer, poly (d,l-lactide) (PDLLA) was dissolved in the above solvent at a final concentration of 3.3% *w*/*w*. An amount of doxy corresponding to 10% *w*/*w* of the total polymer content was dissolved in the core system. The composition of fabricated nanofibers is described in Table 1. Before electrospinning, it was ensured that the polymer solutions were without air and polymer debris. 

### 2.3. Fabrication of Core and Core-shell Type Nanofiber

**Scheme 1:**
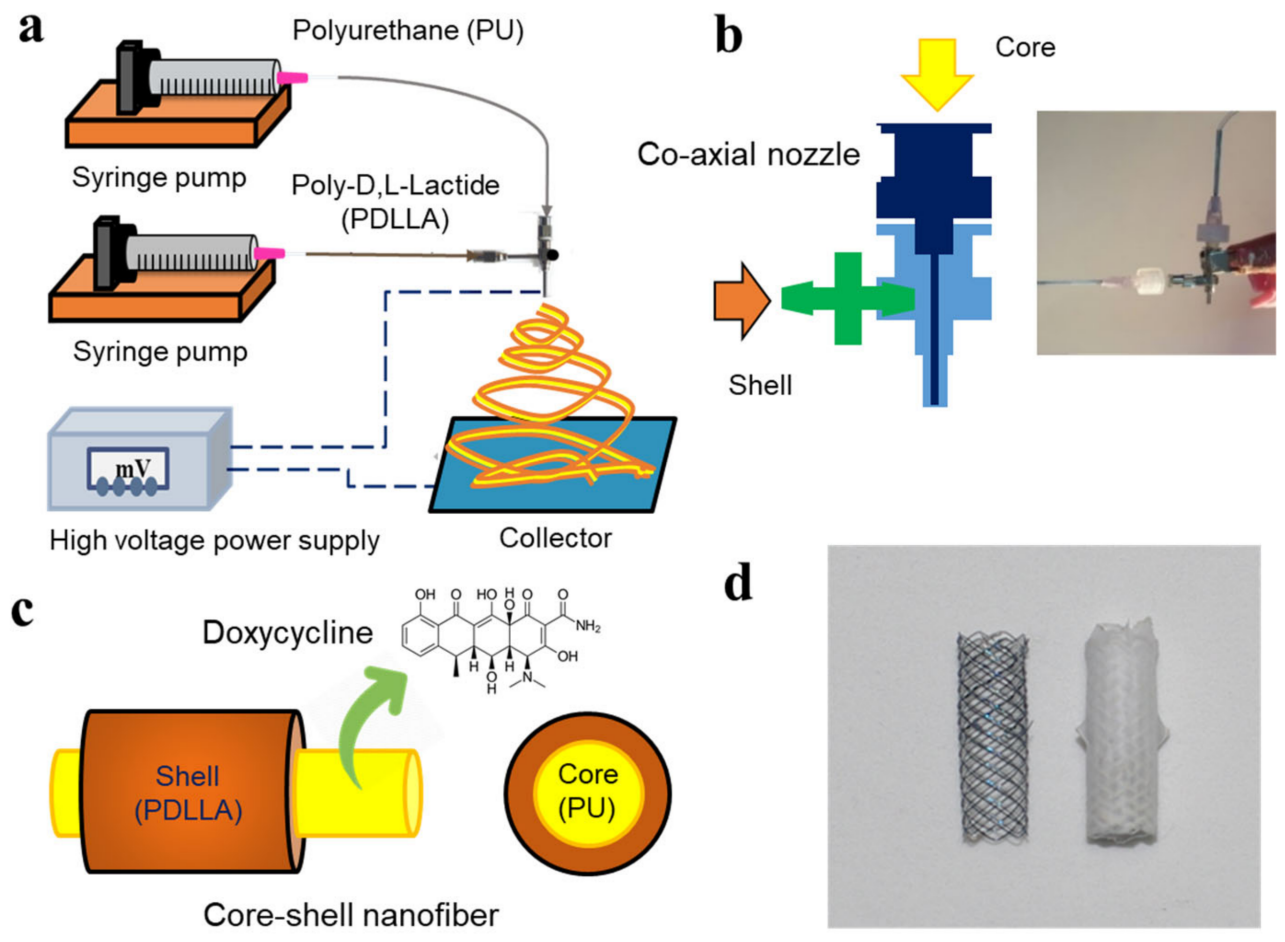
Fabrication of core-shell electrospun nanofiber. (**a**) Schematic diagram of core-shell electro-spinning nanofiber generating system. (**b**) Design of co-axial nozzle for core-shell nanofiber generation. (**c**) Schematic structure core-shell nanofiber (Doxy eluting PU(polyurethane) core and release controlling Poly(d,l-lactide) shell. (**d**) Self-expanding bare metal stent (left) and doxy-eluting PU–PDLLA core-shell electrospun stent for animal test (the maximum external diameter is 6 mm).

A single nanofiber was fabricated using PU solution and injected through a 17-gauge needle with a flow rate of 0.3 mL/h. The electric voltage was optimized using different conditions to ensure the formation of the Taylor cone (Scheme 1). The single nanofiber was collected onto an aluminum foil or metal stent in a rotating cylindrical collector set to 1000 rpm. For the core-shell nanofiber, the solutions for the core and shell were simultaneously injected with a flow rate of 0.3 and 0.9 mL/h, respectively. Polymer solutions were injected through the combined dual metal nozzle (Scheme 1b, 17-gauge for the shell and 23-gauge for the core). Nanofibers were fabricated using various flow rates for the core and shell solution. The electrospun nanofibers were directly collected onto a grounded metal stent or aluminum foil fixed on a rotating cylindrical collector (Scheme 1d). All the nanofibers were well-dried and stored in an air-tight container for further analysis. 

### 2.4. High-Performance Liquid Chromatography (HPLC)

The amount of doxy was quantified using an HPLC system (Alliance 2690 system, Milford, MA, USA). The nanofibers were dissolved for analysis. Fortis C_18_ column (150 × 4.6 mm × 5 µm) was used, and the mobile phase consisted of methanol and 20 mM of phosphate buffer (60:40% *v*/*v*) adjusted to pH 6.0. The mobile phase was fixed at a 1 mL/min flow rate, and the injection volume was 50 µL. The column temperature was maintained at 25 ± 2 °C, and the chromatogram was observed using a UV detector at 262 nm. The calibration was carried out using a standard doxy sample. All the standard and test samples were well-stored until analysis. 

### 2.5. Scanning Electron Microscopy (SEM)

The morphological structure of the nanofibers was analyzed using scanning electron microscopy (SEM). The nanofiber was collected on a stent using a rotating cylinder. The nanofiber-covered stent was dried in a desiccator for 24 h. After platinum coating of the nanofiber scaffold stents, the surface morphology was observed using SEM with an accelerating voltage of 5 kV. 

### 2.6. Identification of the Core-shell Structure 

Fluorescence microscope images of nanofibers were observed to identify the core-shell structure of the nanofiber. Nile red and DAPI were added to the PU core solution and PDLLA shell solution, respectively, and the core-shell nanofiber was electrospun for fluorescence microscope imaging. 

The morphology of the core-shell layer of the nanofibers was further identified using transmission electron microscopy (TEM) (Philips CM200 FEG, FEI Company, Eindhoven, The Netherlands). The single and coaxial electrospinning nanofiber samples were directly collected on the carbon-coated square-grid membrane. The samples were dried at room temperature, observed using TEM at 100 kV, and analyzed with Gatan microscopy suite software 3.0 (Gatan Inc., Pleasanton, CA, USA).

### 2.7. Doxycycline Release Study

The in vitro drug release from the single and core-shell nanofiber was estimated by the conventional method. The fabricated nanofibers were weighed and inserted into a conical tube containing buffer solution adjusted to pH 7.4. The samples were kept on a rotating shaker set to 80 rpm. The temperature was set at 37 ± 2 °C to mimic body conditions. The samples were withdrawn at predetermined times of 0, 1, 3, 7, and 24 h. Whole release media was removed and replaced with equilibrated fresh media at each time point. Samples were centrifuged for 10 min at 4000 rpm after sonication. The supernatant solution was further diluted with methanol and injected for HPLC. All the samples were stored at 4 °C until analysis.

### 2.8. Growth of Fibrosarcoma Cells on the Nanofiber Membrane

Cell growth on the nanofiber membrane was estimated using the HT1080 fibrosarcoma cell line. Cells were incubated with RPMI media containing 10% fetal bovine serum with penicillin and streptomycin. Cells were sub-cultured and recovered for cultivation on the nanofiber membranes. Around 50 × 10^4^ of cells were layered on the doxy-releasing core-shell nanofiber membranes and incubated at 37 °C with 5% CO_2_ for 72 h. After the incubation, the proliferation of cells was observed after DAPI staining using a fluorescence microscope (Axio Observer A1, Zeiss, Oberkochen, Germany). 

### 2.9. Quantitative Real Time Polymerase Chain Reaction (PCR) Estimation 

mRNA levels of MT1-MMP, MMP2, and MMP9, which are representative genes of fibrotic tissue formation [24] were estimated using quantitative real time PCR. The total RNA was isolated from the cells using TRIzol reagent using the standard protocol (Invitrogen, Waltham, MA, USA), and the concentration was determined at 260/280 nm absorbance using a spectrophotometer. First-strand cDNA was generated using a reverse transcription kit (TaKaRa Bio Inc., Kusatsu, Japan) following the manufacturer’s instructions. The expression of mature mRNAs and their target genes was measured as previously reported [25]. Expression levels of target genes were calculated with GAPDH used as an internal standard. The PCR primer sequences are shown in Table S1. 

### 2.10. Statistical Analysis

The data are expressed as the mean ± standard deviation (S.D.). The statistically significant differences were tested using unpaired and one-sided *t*-tests. A *p* value less than 0.05 or 0.01 was considered significant.

## 3. Results and Discussion

Tracheal stenosis is an obstruction of the trachea air passageway that causes breathing problems. Surgical resection of the obstructive hyperplasia is the first treatment option for laryngotracheal stenosis, and stent intubation can be another palliative treatment. However, surgical resection of an area greater than 4 cm is associated with an increase in the rate of failure and may result in fibrotic tissue formation in the resected area [26,27]. Postintubation fibrosis is another problematic complication in patients. Most cases require proper wound healing treatment for the prevention of fibrotic tissue formation. Among the therapeutic approaches, the use of doxy may be the most attractive treatment option. Hence, we hypothesized that a doxy-loaded nanofiber stent may be a treatment option for tracheal stenosis.

We fabricated a doxy-eluting PU core-PDLLA shell nanofiber-covered stent using the electrospinning technique. PU and PDLLA are biocompatible and are the most commonly used polymers in nanofiber manufacturing [28]. PU is enzymatically stable over its lifetime without serious interaction with organ tissues and is widely used as a covering membrane for gastrointestinal stents because of its mechanical strength [29]. PDLLA has been used to manufacture nanofibers for controlled drug release [30,31]. In our study, the doxy-releasing nanofiber as a covering membrane of the trachea stent was successfully fabricated using PU as the core polymer and PDLLA as the outer shell polymer (Scheme 1d). 

**Figure 1:**
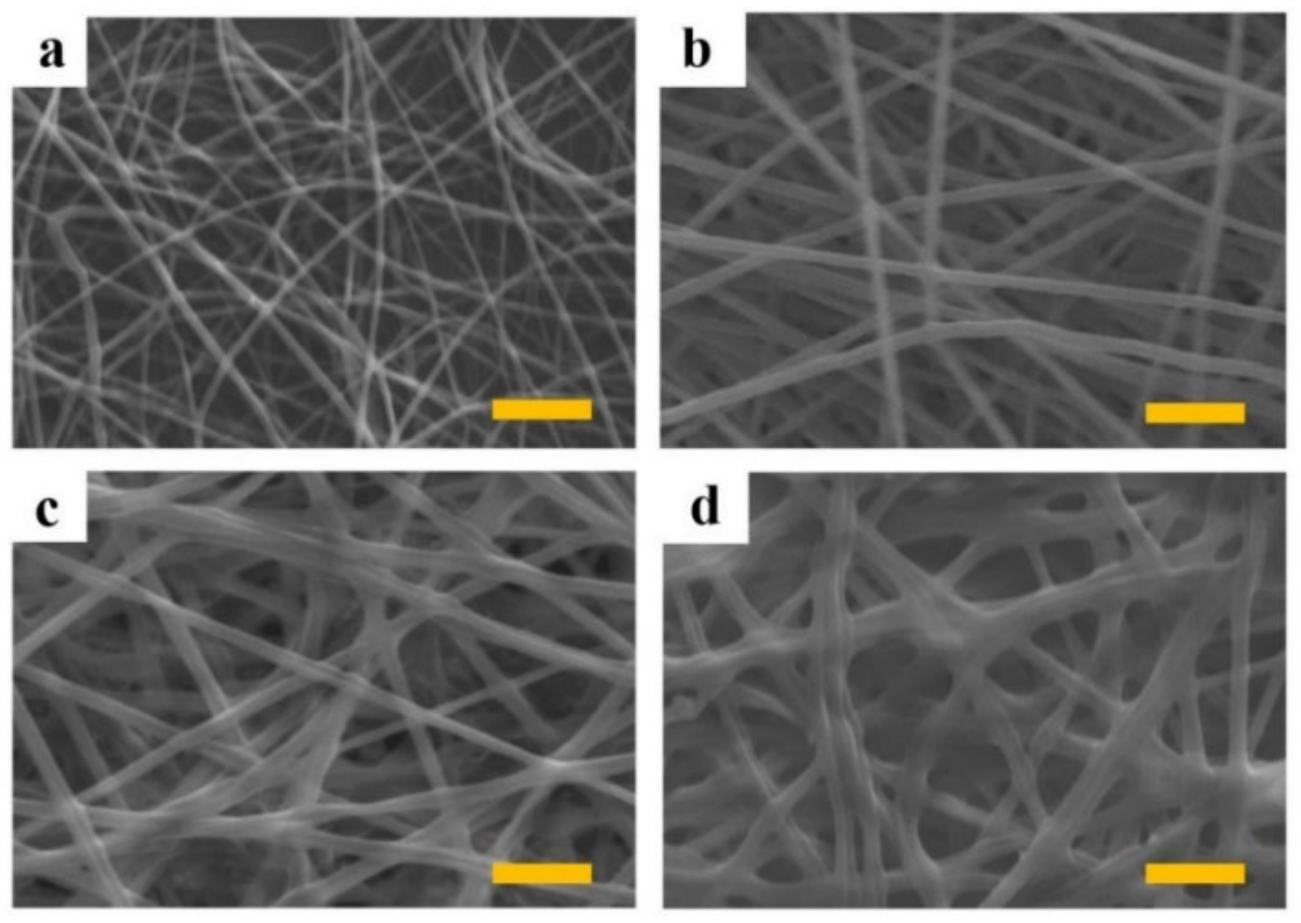
Scanning electron microscopic images of electrospun polyurethane (PU) single nanofiber (**a**) and PU core-PDLLA shell (1:1. 1:2 and 1:4) electrospun nanofiber (**b**–**d**). Yellow scale bar represents 5 μm.

The morphology and corresponding diameters of the nanofibers were examined using microscopic imaging technologies (SEM, TEM, and fluorescence microscope). The SEM images showed that the single nanofiber is thin compared with the core-shell nanofiber (Figure 1). The observed diameter of single and core-shell (1:1, 1:2 and 1:4) nanofiber was 0.39 ± 0.07, 0.71 ± 0.09, 1.71 ± 0.27 and 2.65 ± 0.93 µm, respectively (Table 1). Generally, the fabricated nanofibers had a smooth surface, narrow structure, and uniform distribution.

**Figure 2:**
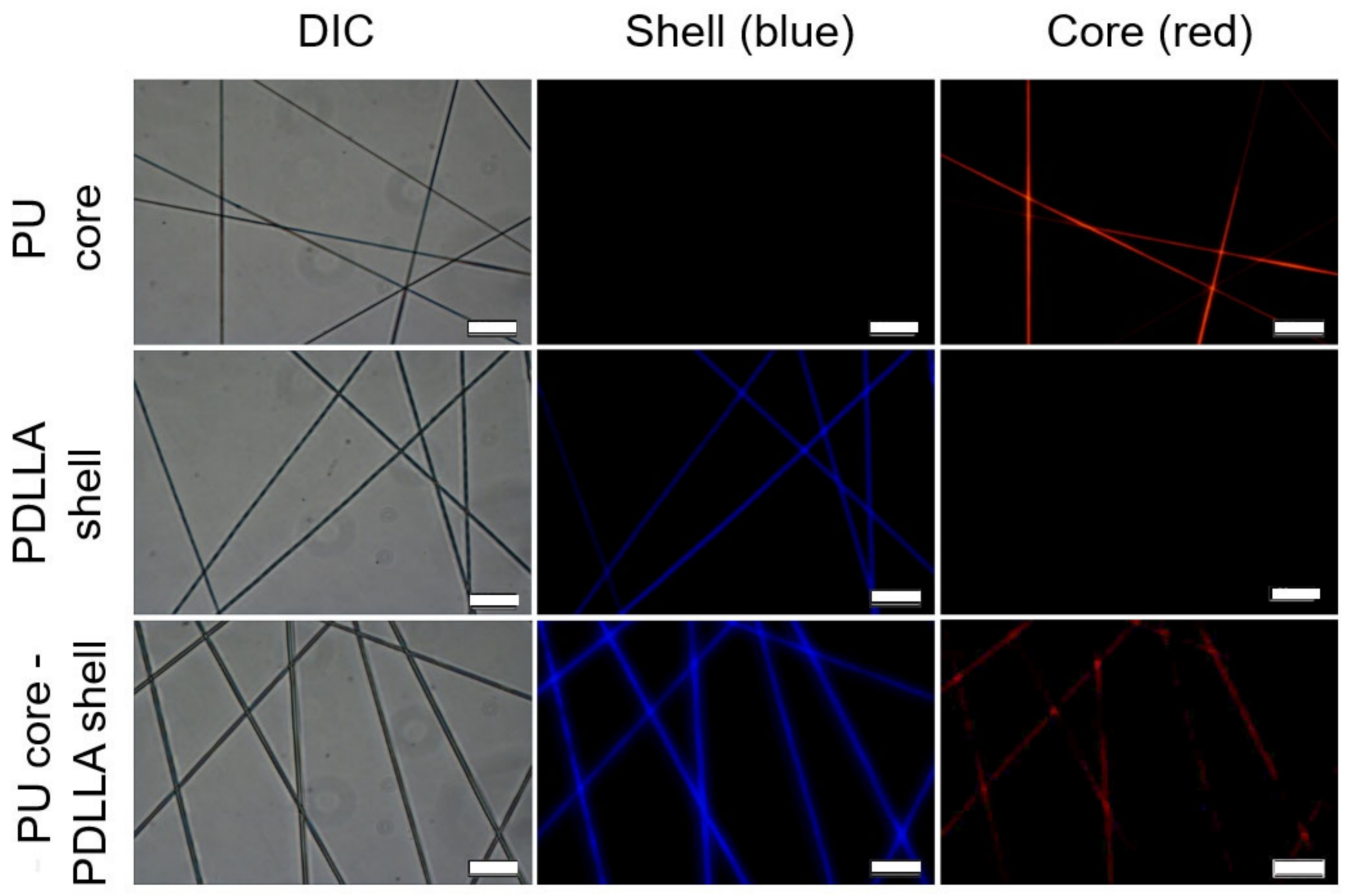
Fluorescence microscopic images of nanofibers; images of single nanofiber of PU core (top, red fluorescence), PDLLA shell (middle, blue fluorescence) and PU core–PDLLA shell nanofiber (bottom, red fluorescence for core and blue fluorescence for shell). Blue and red fluorescence were acquired from 4′,6-diamidino-2-phenylindole (DAPI) and Nile red, respectively. The scale bar represents 20 μm.

**Figure 3:**
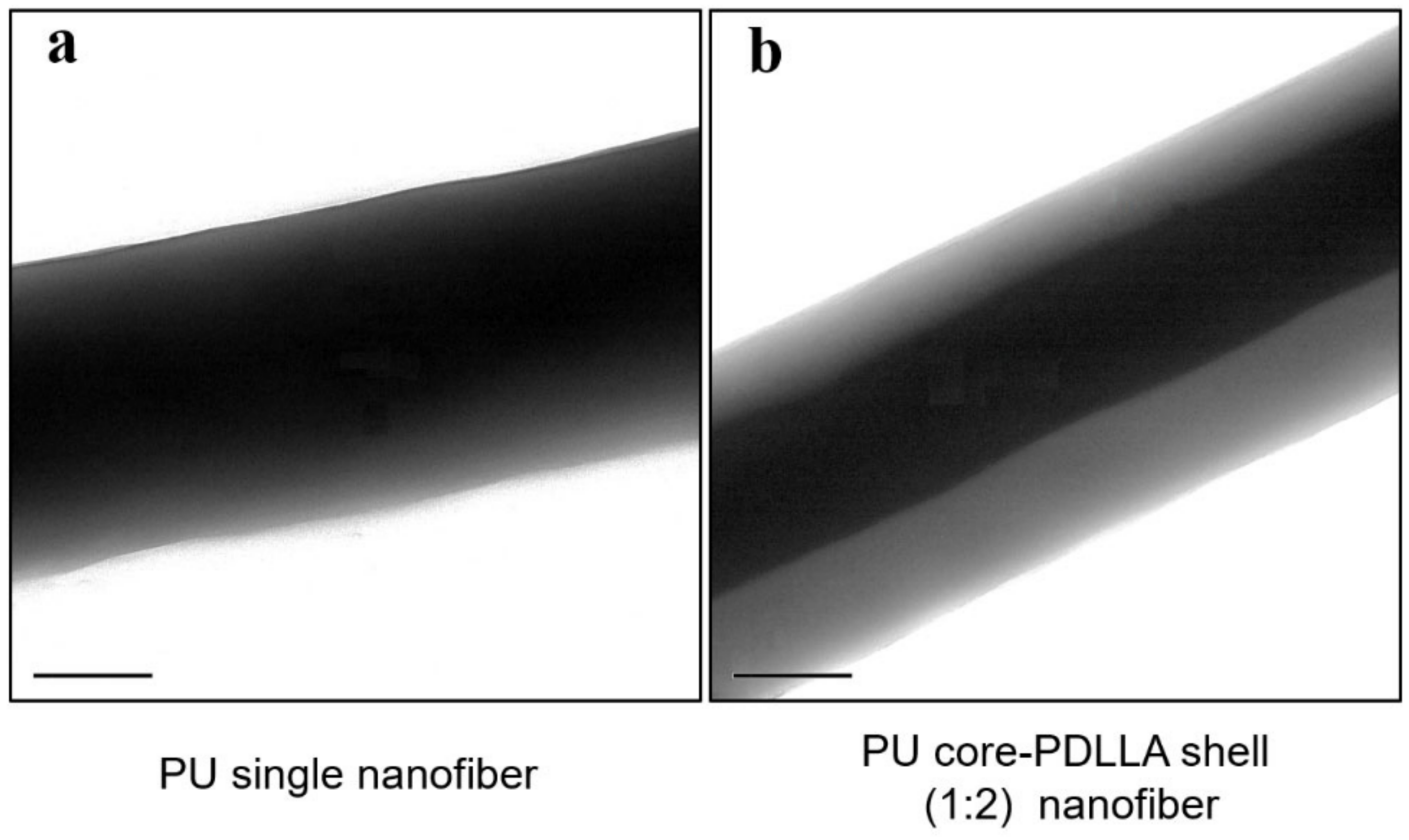
Transmission electron microscopic images of single and core-shell (1:2) electrospun nanofibers. (**a**) PU single nanofiber. (**b**) PU core–PDLLA shell (1:2) nanofiber. Thickness of single nanofiber layer and core-shell nanofiber was around 0.51 µm and 0.47 µm (0.25 µm for core and 0.11 µm for shell) in the TEM images, respectively. The scale bar represents 0.2 μm.

Fluorescent images of nanofibers confirmed the successful formation of core-shell structure (PU core with blue fluorescence and PDLLA shell with red fluorescence) (Figure 2). TEM images allowed for the thickness estimation of mono and core-shell layers of fabricated nanofibers (Figure 3). Single nanofibers had a smooth surface without interruption. The core-shell image showed the layer of the outer and inner part. We optimized the core-shell structure by adjusting the polymer concentration and the flow rate of the injecting solution (Table 1). 

The loading efficiency was estimated using the HPLC after complete dissolution of drug from nanofiber membrane in organic solvent. The result is shown in Table 1. Data show core-shell nanofiber higher entrapment efficiency (103.8 ± 1.0 for 1:2 nanofiber and 105.0 ± 4.9 for 1:4 nanofiber) than single nanofiber (89.9 ± 5.3). 

**Figure 4:**
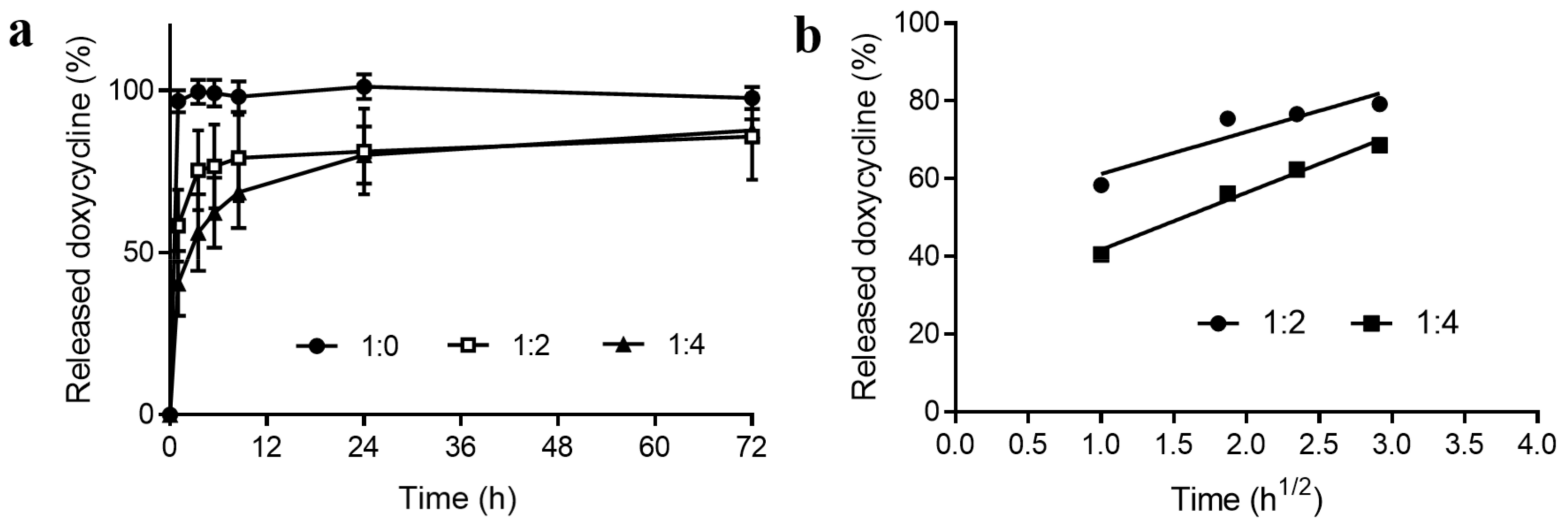
The in vitro drug release from the single and core-shell nanofiber was estimated by the conventional method. (**a**) Release profiles of doxy from single (1:0) and core-shell (1:2, 1:4) nanofibers in phosphate buffer adjusted to pH 7.4. The release medium was maintained at 37 °C. The amount of doxy was measured by HPLC (high performance liquid chromatography). (**b**) Higuchi modeling of doxy release from core-shell (1:2, 1:4) nanofibers. Solid lines are a regression curve for the Higuchi equation. r^2^ and other statistical values are listed in figure.

The in vitro release profile of the doxy is shown in Figure 4. An initial burst release was observed for all nanofiber compositions. The single (1:0) nanofiber released 96.7 ± 3.4% of the loaded doxy within 1 h of the release study. The core-shell (1:2) nanofiber exhibited 58.4 ± 11.1% doxy release at 1 h, and the release amount increased to 81.2 ± 13.3% at 24 h. Rapid release is attributed to the hydrophilic nature of doxy (doxycycline hyclate is highly water soluble up to 50 mg/mL). The initial release amount of doxy from the core-shell (1:4) nanofiber was 40.6 ± 10.0% at 1 h. A subsequent delayed release up to 80.1% for 24 h was observed. The PDLLA shell layer of the core-shell nanofiber at a 1:4 ratio functioned as a barrier layer to doxy release. However, other nanofibers (PU only, 1:1. 1:2 and 1:3 core-shell nanofiber) showed rapid release at the initial stage and non-release during the last stage. We postulated that the in vitro release study performed in the buffer solution does not adequately reflect the intra-trachea environment. In clinical trials, the doxy-releasing nanofiber stent is expected to be anchored inside of the trachea tube and make direct contact with the sticky mucus layer of the trachea [32]. Therefore, the release of doxy after tracheal intubation must be delayed more than what was observed at the in vitro study. This observation was well presented in previous studies. Schwartz et al. reported in vitro release of everolimus from the cardiovascular stent reached a maximum at 10 days. However, the in vivo study showed release was extended up to 180 days [33]. Sustained release of doxy from nanofibers can efficiently inhibit fibrosis and the formation of hyperplasia tissues [17].

In addition, we applied different drug release models to our study (Figure 4b and Table S2). Single fiber doxy release is too instant that its profile did not match with zero order, first order, Higuchi, Korsmeyer–Peppas, and Hixson–Crowell models. However, a part of the core-shell fibers release data (1:2 and 1:4) shows consistency with some models. The applied data range is from 1 to 8.5 h for both compositions. The model-data correspondences were evaluated by correlation coefficient (r^2^), and root-mean-square error (RMSE). As a result, for all models, 1:4 composition features higher r^2^ whereas RMSE were lower. Particularly, the r^2^ records the highest on the Higuchi model with the minimum RMSE value. A higher r^2^ in the Higuchi model indicates that the drug’s diffusion across the matrix gets more dominant in the 1:4 composition fiber (r^2^: 0.9851). Lower RMSE (1.2735) supports that the 1:4 sample is closer to the assumption of the Higuchi model that the initial drug concentration in the matrix is much higher than drug solubility. As hydrophilic PDLLA portion increases, doxy can reside well inside the matrix. Therefore, diffusion of the drug across PDLLA may become a meaningful mechanism in the fiber’s drug release manner.

**Figure 5:**
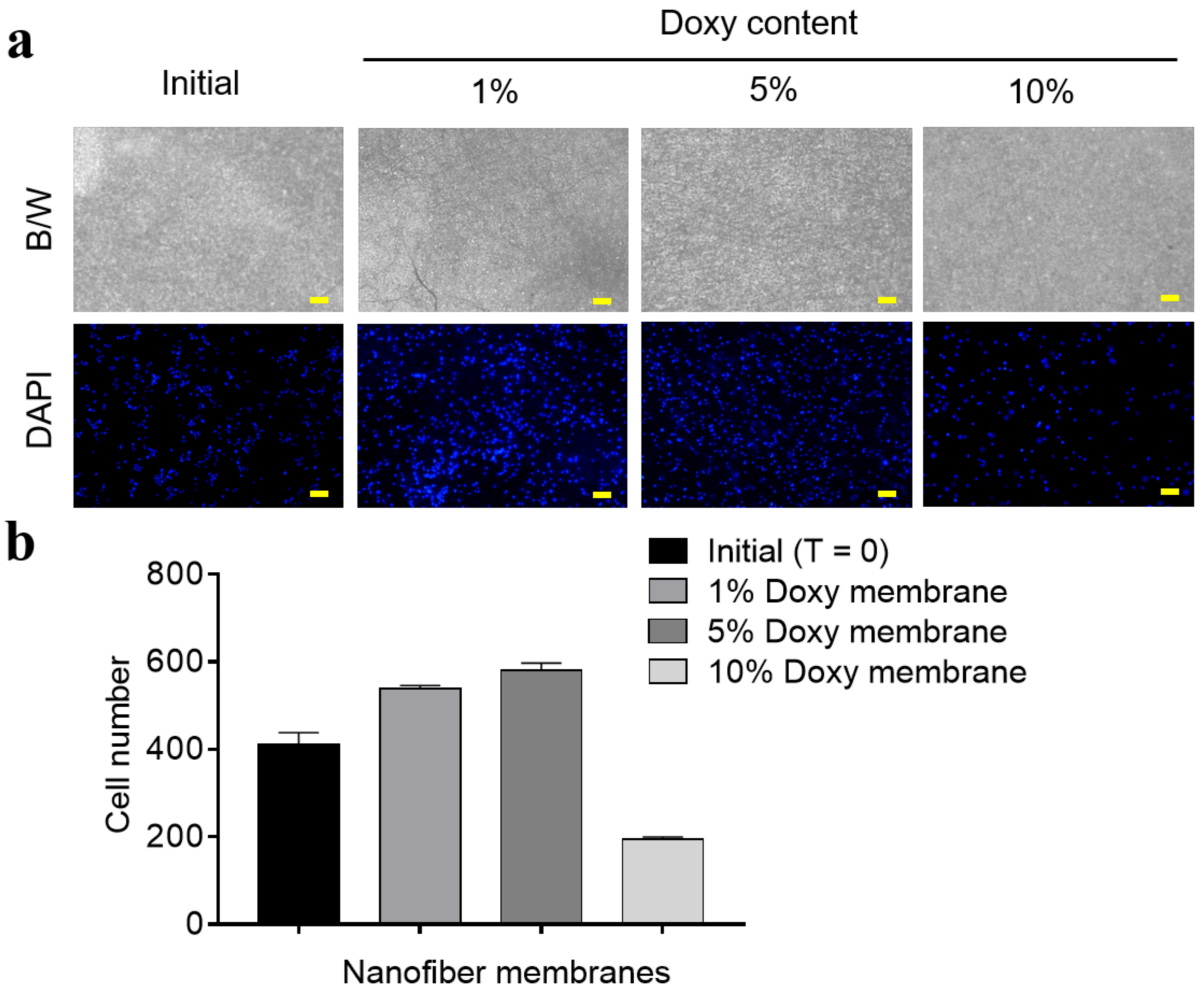
Cell growth on the doxy-releasing nanofiber membrane. (**a**) HT1080 mouse fibrosarcoma cells were placed on the nanofiber membrane of different doxy concentration, incubated for 72 h and stained with DAPI for fluorescence microscopy (Logos biosystems, Gyeonggi-do, Korea). (**b**) Number of HT1080 cells after 72 h of incubation on the top of the doxy-eluting membrane. Number of cells was calculated using Image J software. Error bars indicate SEM (*n* = 3). The scale bar represents 50 μm.

**Figure 6:**
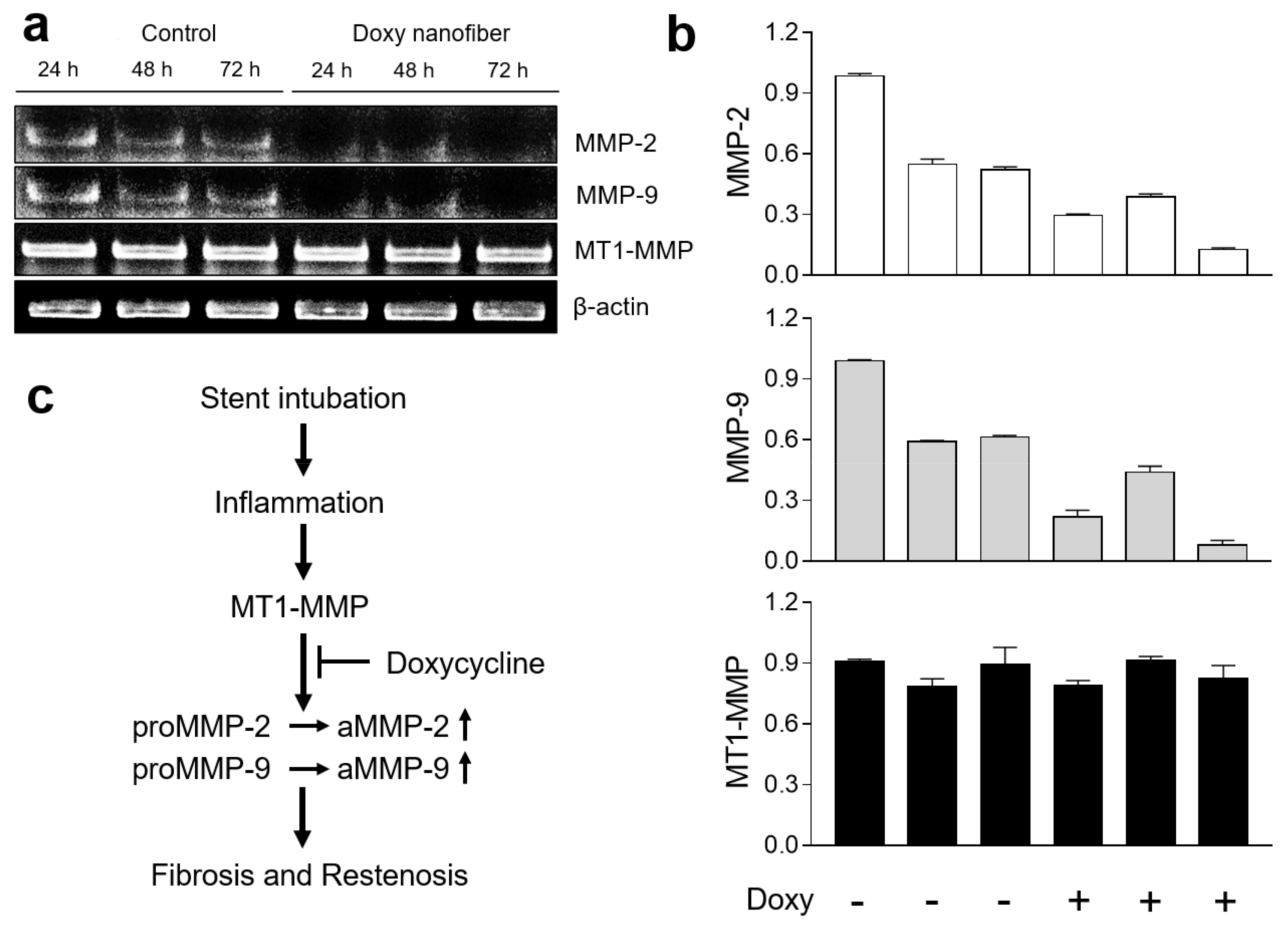
Effect of doxy-releasing nanofibers on MMP expression. HT1080 mouse fibrosarcoma cells were placed on the membrane incubated for 24, 48 and 72 h. (**a**) Real-time PCR analysis shows MMP-2, MMP-9 and MT1-MMP mRNA levels. (**b**) Relative MMPs activities were quantified in image J software. Doxy-releasing nanofiber inhibits mRNA levels of the MMP-2 and MMP-9. (**c**) General molecular pathway of MMP expression and resulting tissue fibrosis after a stent intubation. Error bars indicate SEM (*n* = 3).

We studied the growth of fibrosarcoma cells on the nanofiber membranes and the results are shown in Figure 5. DAPI staining proved that doxy-releasing nanofiber inhibits growth of fibrosarcoma cells in a dose-dependent manner. In the 10% doxy-releasing nanofiber, cell growth inhibition was observed. Those results were correspondent with further study results of MMP expression estimation on in vitro and in vivo models (Figure 6 and Figure S1). Degradation of core-shell nanofiber after the dissolution study or cellular disposition study was not observed in this study. Nanofiber degradation was studied by many scientists. Specifically, Kim et al. reported that the PU nanofiber co-incubated with cells partly lost its fiber shape after three days [34]. In any case, nanofiber after the tissue intubation will be degraded and washed out in the body. In our case, we assume hydrophobic PDLLA shell largely affects the nanofiber integrity and maintains the initial integrity of fiber for the full release of doxy after a tracheal intubation. 

The in vitro cell study using HT1080 suggested that the doxy-releasing nanofiber might display promising effects for the treatment of tracheal stenosis by inhibiting mRNA expression of MMP-2 and MMP-9. Paradoxically, stent-intubation causes chronic inflammation, which induces over-expression of MMP-2 and MMP-9 and finally fibrotic re-stenosis of the trachea. Biologically, MT1-MMP plays a crucial role in the proteolytic activation of MMP-2 and MMP-9. Our study showed that the doxy-releasing nanofiber inhibited MMP-2 and MMP-9 but not MT1-MMP activity (Figure 6). A slight depletion of MT1-MMP was observed due to doxy over the time period studied. However, the in vivo study showed total inhibition of MMP-2, MMP-9 and MT1-MMP (Figure S1). These results suggest that the doxy-loaded nanofiber may prevent fibrosis via an MMPs inhibition mechanism. 

## 4. Conclusion

In this study, we successfully developed a doxy-eluting core-shell nanofiber for the prevention of fibrosis, which frequently occurs after trachea stent intubation. Morphology characterization using SEM, TEM, and fluorescence imaging indicated the successful formation of a core-shell structured nanofiber. The in vitro release study results revealed that adjusting the compositional ratio of the polymeric shell affected the release pattern of doxycycline. The cellular study proved that the doxy-eluting nanofiber inhibited the expression of MMP-2 and MMP-9. Our results suggest that a doxy-releasing nanofiber stent can prevent fibrotic deformation of tracheal tissues after stent intubation. 

## Figures and Tables

**Table 1:** Electrospinning condition and composition of single and core-shell nanofiber.

Type	PU Core ^1)^		PDLLA Shell ^2)^		Nanofiber Thickness (μm, mean ± S.D.)	Doxycycline	
	Flow Rate (mL/h)	Polymer Weight (mg) ^3)^	Flow Rate (mL/h)	Polymer Weight (mg) ^3)^		Applied Amount for Spinning (mg)	L.E. (%, mean ± S.D., *n* = 3) ^4)^
Single (1:0)	0.3	30	-	-	0.39 ± 0.07	3	89.9 ± 5.3
Core-shell (1:1)	0.3	30	0.9	30	0.71 ± 0.09	6	97.1 ± 6.2
Core-shell (1:2)	0.3	30	1.8	60	1.71 ± 0.27	9	103.8 ± 1.0
Core-shell (1:4)	0.3	30	3.6	120	2.65 ± 0.93	15	105.0 ± 4.9

^1)^ 10% of the PU (polyurethane) solution was used for core-forming electrospinning. ^2)^ 3.3% of the poly(d,l-lactide) solution was used for shell-forming electrospinning. ^3)^ Theoretical disposition amount (mg) of core and shell forming polymers after the electrospinning. ^4)^ L.E. stands for drug loading efficiency.

## Supplementary Materials

The following are available online at https://www.mdpi.com/1999-4923/11/8/421/s1, Figure S1. Tissue histology and mRNA level of MMPs in HT1080 fibrosarcoma xenograft models. The tumors after 2 weeks of subtumoral membrane implantation (blank nanofiber membrane and doxy-eluting nanofiber membranes) were recovered for mRNA assay (a) and histological observation (b,c). Table S1. The primer sequence of MMPs for RT-PCR study. Table S2. Statistical values for mathematical modeling of the kinetics of release for core-shell fibers (1:2 and 1:4)
