# Supplementary material for: Doxycycline-Eluting Core-Shell Type Nanofiber-Covered Trachea Stent for Inhibition of Cellular Metalloproteinase and Its Related Fibrotic Stenosis
Source: Pharmaceutics. 2019 Aug 19;11(8):421. doi: 10.3390/pharmaceutics11080421 (PMC6723391; doi:10.3390/pharmaceutics11080421)
Supplement: Supplementary file 1 [file pharmaceutics-11-00421-s001.pdf]

pharmaceutics

MDPI

Supplementary Materials: Doxycycline-Eluting Core-Shell Type Nanofiber-Covered Trachea Stent for Inhibition of Cellular Metalloproteinase and Its Related Fibrotic Stenosis

Rengarajan Baskaran, Un-Jeong Ko, Enkhzaya Davaa, Ji-Eun Park, Yixin Jiang, Junghan Lee and Su-Geun Yang *

Tissue MMP inhibition of doxy-releasing nanofiber

All animal care and experimental procedures were conducted in accordance with the guidance for Experimental Animal Research Committee of Inha University. HT1080 fibrosarcoma cells (5.0×10^6 cells in 100 μL) were subcutaneously injected on the flank side of female BALB/C nude mice (Orient Co., Korea). When the tumors grew to an average diameter of 10 mm, 10% doxy containing nanofiber membranes and blank membranes were sub-tumorally implanted under the tumor. After 2 weeks of implantation, tumor tissues were recovered and subjected to H&E histological assay and MMP RNA assay.

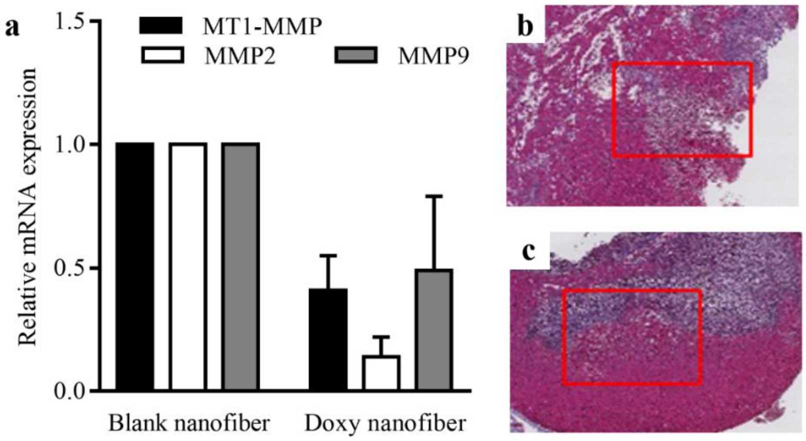

Figure S1. Tissue histologies and mRNA level of MMPs in HT1080 fibrosarcoma xenograft models. The tumors after 2 weeks of sutumoral membrane implantation (blank nanofiber membrane and doxy-eluting nanofiber membranes) were recovered for mRNA assay (**a**) and histological observation (**b** and **c**).

Table S1. The primer sequence of MMPs for RT-PCR study.

Gene	Primers (5′-3′)	Product Size (bp)
MMP-2	Forward: GGCCCTGTCACTCCTGAGAT<br>Reverse: GGCATCCAGGTTATCGGGGA	120
MMP-9	Forward: CAACATCACCTATTGGATCC<br>Reverse: CGGGTGTAGAGTCTCTCGCT	100
MT1-MMP	Forward: GCTTGCAAGTAACAGGCAAA<br>Reverse: AAATTCTCCGTGTCCATCCA	589
β-actin	Forward: GGAAATCGTGCGTGACATTAAGG<br>Reverse: GGCTTTTAGGATGGCAAGGGAC	700

Table S2. Kinetic modeling on doxycycline release from core-shell fibers (1:2 and 1:4).

Core-Shell (1:2)				
	r^2	RMSE	Slope	Intercept
Zero order	0.7376	4.2090	2.5685	60.544
First order	0.7898	0.0531	−0.0375	1.5972
Higuchi	0.8552	3.1264	10.828	50.412
Korsmeyer-Peppas	0.8520	0.3533	2.44	0.0063
Hixson-Crowell	0.7248	0.0865	0.0511	−0.7161

Core-Shell (1:4)				
	r^2	RMSE	Slope	Intercept
Zero order	0.9258	2.8398	3.651	40.066
First order	0.9681	0.0182	−0.0364	1.7903
Higuchi	0.9851	1.2735	14.744	26.979
Korsmeyer-Peppas	0.8688	0.3430	2.5406	−0.1587
Hixson-Crowell	0.8988	0.0785	0.0851	−1.2034
